# The Neglected Internodal Tract—A Cardiac Conduction System Structure Homologous to the Development and Regulation of the Sinoatrial Node

**DOI:** 10.31083/RCM27882

**Published:** 2025-04-22

**Authors:** Yuanqin Zhao, Juyi Wan, Bin Liao, Man Qi

**Affiliations:** ^1^State Key Laboratory of Cardiovascular Disease, Fuwai Hospital, National Center for Cardiovascular Diseases, Chinese Academy of Medical Sciences and Peking Union Medical College, 100037 Beijing, China; ^2^Department of Cardiovascular Surgery, The Affiliated Hospital, Southwest Medical University, Metabolic Vascular Diseases Key Laboratory of Sichuan Province, Key Laboratory of Cardiovascular Remodeling and Dysfunction, 646000 Luzhou, Sichuan, China; ^3^Key Laboratory of Medical Electrophysiology, Ministry of Education & Medical Electrophysiological Key Laboratory of Sichuan Province (Collaborative Innovation Center for Prevention of Cardiovascular Diseases), Institute of Cardiovascular Research, Southwest Medical University, 646000 Luzhou, Sichuan, China; ^4^Chinese People’s Liberation Army (PLA) General Hospital, College of Pulmonary & Critical Care Medicine, 100091 Beijing, China; ^5^Department of Cardiology, Chinese PLA General Hospital, 100853 Beijing, China; ^6^State Key Laboratory of Cardiovascular Diseases and Medical Innovation Center, Shanghai East Hospital, School of Medicine, Tongji University, 200120 Shanghai, China

**Keywords:** cardiac conduction system, internodal tracts, sinoatrial node, arrhythmia, transcription factors

## Abstract

The existence of internodal tracts (ITs) is controversial. Indeed, ITs in the cardiac conduction system (CCS), connected to the sinoatrial node (SAN), transmit electrical signals quickly to the left atrium and the atrioventricular node (AVN). Interestingly, research has suggested that the ITs and the tail of the SAN may share developmental homology. Additionally, many studies indicate that ITs blockage can lead to atrial conduction block and is associated with atrial fibrillation (AF). However, few studies have been reported on the morphogenesis, development, and function of ITs. Therefore, this paper aims to review the morphogenesis, development, and function of ITs, focusing on the regulatory mechanisms of transcription factors (TFs), such as NK2 homeobox 5 (NKX2.5), SHOX homeobox 2 (SHOX2), hyperpolarization activated cyclic nucleotide gated potassium channel 4 (HCN4), and T-box transcription factor 3 (TBX3) in the development and morphogenesis of ITs. This review also explores the causes of arrhythmias, especially atrial block, in order to provide new insights into the pathogenesis of CCS disorders.

## 1. Introduction

**Fig. 1.**
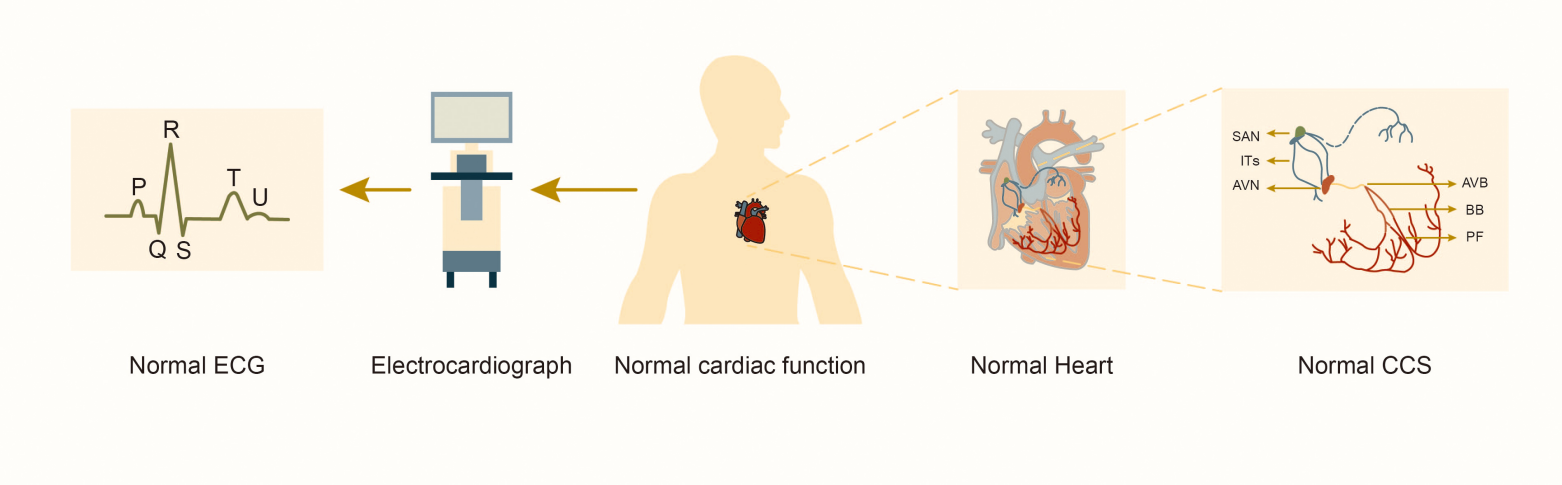
**Structure and function of the normal cardiac conduction system**. Abbreviations: SAN, sinoatrial node; ITs, internodal tracts; AVN, atrioventricular node; AVB, atrioventricular bundle; BB, bundle branches; PF, Purkinje fibers; CCS, cardiac conduction system; ECG, electrocardiograph.

According to the Centers for Disease Control and Prevention (CDC), more than 600,000 people in the United States die annually from heart failure or sudden cardiac death [1]. Approximately 50% of patients with heart diseases die from arrhythmias [2]. Studies also revealed that nearly 50% of patients undergoing congenital heart surgery developed arrhythmias after surgery [3]. Arrhythmia mechanisms include abnormalities in impulse formation and/or conduction, among which abnormalities in impulse conduction often present as various conduction blocks, including sinoatrial, atrial, atrioventricular, and ventricular blocks. The cardiac conduction system (CCS) generates and transmits impulses to maintain the heart’s rhythmic beats. CCS also consists of specialized myocardial cells that coordinate the contractions of the atria and ventricles to establish the heart’s rhythm. Thus, CCS is essential in the formation and normal function of the heart (Fig. 1). Consequently, the dysfunction of any component can lead to serious heart conditions, such as arrhythmias, decreased cardiac output, and even sudden death. Although noted as key components in CCS, the existence, morphogenesis, development and function of internodal tracts (ITs) have rarely been reported systematically. Actually, recent studies have increasingly confirmed the existence of ITs, although their specific anatomy still require further investigation [4, 5]. Studies also indicate that functional block of ITs can cause clinical manifestations such as atrial conduction block or atrial fibrillation (AF) [6]. Therefore, this article aims to review the structural composition and development of ITs in CCS as well as transcription factors (TFs) playing regulatory roles in ITs’ development for the purpose of laying groundwork for further research into the molecular mechanisms of atrial block and AF and also providing new directions for treating arrhythmias.

## 2. The Development of Internodal Tracts in Cardiac Conduction System

The CCS consists of ITs with fast conduction, sinoatrial node (SAN), atrioventricular node (AVN) with slow conduction and specialized myocardial cells such as the atrioventricular bundle (AVB, or “His bundle”), left and right bundle branches, and Purkinje fibers (Fig. 1). These cells coordinate the rhythmic contractions of the atria and ventricles to establish the heart’s rhythmic beating. Studies of lineage tracking and electrophysiological characteristics of CCS cells reveal that these cells don’t belong to the same type. Different cell types exhibit significant heterogeneity in morphology, function and molecular characterization [7]. SAN in the right atrium initially generates electrical pulse, depolarizing adjacent atrial myocardial cells before rapidly propagating through ITs to AVN and the left atrium. AVN delays impulse transmission, slowing the electrical pulse speed by approximately 0.04 seconds. This delay allows the atrium to contract and pump blood into the ventricle before it contracts, ensuring the sequential contraction of the atrial and ventricular muscles and adapting to intracardiac blood circulation. Subsequently, the electrical pulse rapidly travels through the atrioventricular bundle to the left and right bundle branches and the Purkinje fiber network, activating ventricular myocardial contractions [8].

**Fig. 2.**
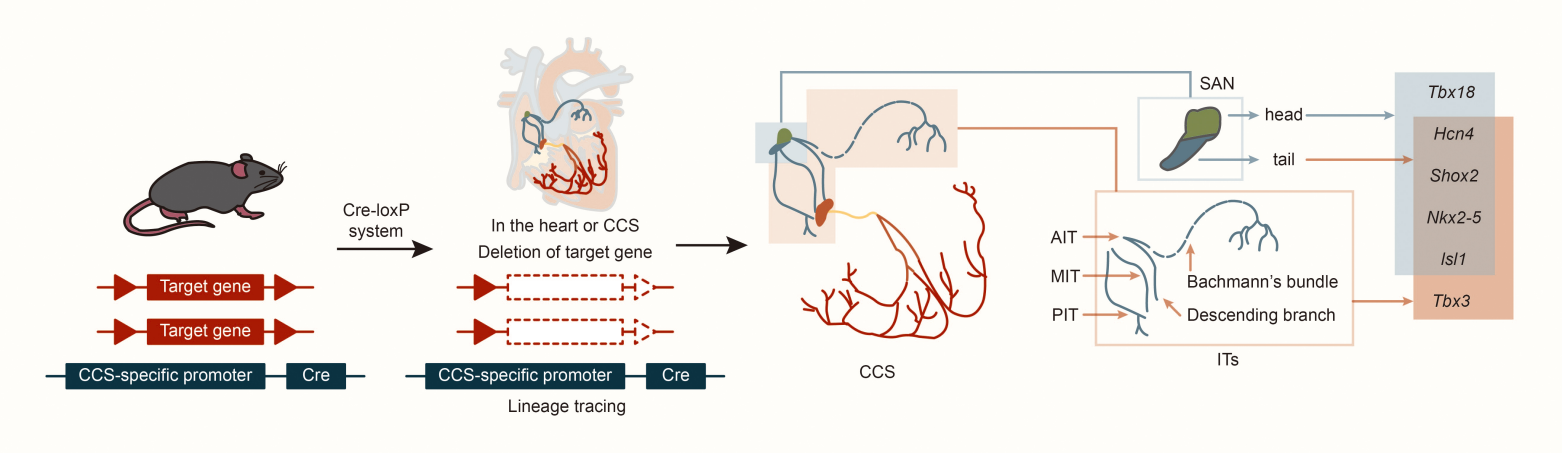
**Structure and lineage tracing of sinoatrial node and internodal tracts**. Abbreviations: Cre, cyclization recombinase; AIT, anterior internodal tract; MIT, middle internodal tract; PIT, posterior internodal tract; *Tbx18*, T-box18; *Hcn4*, hyperpolarization activated cyclic nucleotide gated K^+^ 4; *Shox2*, SHOX homeobox2; *Nkx2-5*, NK2 homeobox 5; *lsl1*, ISL1 transcription factor, LIM/homeodomain; *Tbx3*, T-box 3.

In cardiac development, the first heart field (FHF) and the second heart field (SHF) are critical embryonic regions derived from distinct populations of cardiac progenitor cells. The temporal sequence of their involvement defines their contributions to the cardiac lineage. The FHF primarily contributes to the formation of the left ventricle (LV), whereas the SHF promotes the right ventricle (RV) and outflow tract (OFT). Both fields also play essential roles in developing the atria and septum [9]. Using specific markers such as *Hcn4* (hyperpolarization-activated cyclic nucleotide gated K^+^ 4, in FHF), *Isl1 *(ISL1 transcription factor, LIM/homeodomain, in SHF) and *Tbx18* (T-box18, in the venous pole of the posterior heart field), CCS lineage tracing in mice revealed that AVN, His bundle and Purkinje fibers express only *Hcn4*, but not *Isl1* or *Tbx18* [10, 11, 12]. This suggests that the AVN, His bundle, and Purkinje fibers primarily develop from the FHF. Further, experiments tracking *Tbx18* expression revealed that cells in the SAN head express both *Tbx18* and *Isl1*, while the SAN junction/tail and ITs express only *Isl1* [12]. Results also indicate that cells in the SAN head may originate from the posterior heart field in the venous pole of the second heart field, while cells in the ITs and SAN junction originate from the SHF [10, 11, 12]. Studies using CCS–*lacZ* transgenic mice described three ITs connecting the SAN and AVN, as well as the embryonic origins of the SAN and ITs [13, 14]. Subsequent studies on the expression patterns of *Hcn4 *and *Tbx3* (CCS markers) in mouse embryonic heart development suggested that development of ITs may occur simultaneously with the SAN junction [15, 16]. Morphogenesis might begin at embryonic day (E) 9.5 and be completed by E12.5, with *lacZ* and *Tbx3* expression continuing at least until E17.5. Nakashima *et al*. [17] found that NK2 homeobox 5 (*Nkx2-5*, also named *Nkx2.5*) suppresses the proliferation of atrial working myocytes and confines SAN, AVN, and ITs. In addition, other laboratories have found that *Shox2* is expressed as early as E8.5 in mouse embryos on the dorsal side of the primitive cardiac tube; on E9.5, *Shox2* is expressed in the transition zone between the venous sinus and the total atrium; on E10.5, *Shox2* is most strongly expressed in the venous sinus and venous valve; on E11.5, *in situ* hybridization of the entire heart showed that *Shox2* expression expanded to include the SAN, venous valves and ITs [14, 18, 19, 20]. This suggests that ITs and SAN junction development may share similar regulatory mechanisms (Fig. 2).

## 3. The Morphology of Internodal Tracts

There is ongoing controversy regarding the existence of specialized conduction tissues, known as ITs, between the SAN and AVNs in the CCS. Early theories suggested that these conduction bundles were crucial for rapidly transmitting electrical signals [21]. However, due to the low expression of gap junction proteins in these bundles, they were once thought to have little or degraded signal conduction function and were considered less significant in mature hearts [16]. Some researchers argue that electrical signals are transmitted radially along the atrial muscle, relying on the arrangement of myocardial cells and gap junction proteins rather than on specialized conduction bundles. Clinical observations of dynamic electrocardiograms have shown that atrial contraction time is slightly prolonged during sinus excitation compared to ectopic excitation. This has led to skepticism about specialized atrial conduction pathways [22]. However, whether these conduction pathways were because of the presence of specialized conduction tissue or because of the anisotropic orientation of the muscle fibers remains controversial [6].

Nevertheless, numerous electrophysiological and biochemical studies have confirmed the existence of intra-atrial conduction bundles. Certain myocardial cells in the atrium, which differ in the electrophysiological aspect, connect SAN and AVN and are termed ITs [23]. In 1963, Thomas N. James [24] identified three conduction tracts connecting the SAN and the AVN, named the anterior internodal tract (AIT), middle internodal tract (MIT), and posterior internodal tract (PIT), based on their anatomical locations. The AIT, which includes part of the Bachmann bundle (BB), is the most complex (Fig. 2) and originates from the anterior edge of the SAN, extending towards the left front, arching around the superior vena cava (SVC) and the anterior wall of the right atrium. The AIT splits into two bundles: one extends through the atrial sulcus into the left atrium, known as the BB, connecting the left and right atrial walls; the other descends diagonally behind the aortic root to the upper edge of the AVN, known as the descending branch. The MIT, or Wenckebach’s bundle, originates from the posterior upper edge of the SAN, arches to the right, passes behind the superior vena cava, and descends along the right side of the atrial septum into the upper edge of the AVN. The PIT, or Thorel’s bundle, extends from the posterior edge of the SAN along the terminal ridge to the inferior vena cava valve (Eustachian’s ridge), then passes slightly above the coronary sinus to the posterior edge of the AVN before extending rapidly downward into it. During its course, the PIT branches out and spreads along the right atrial comb muscle to the back of the right atrium [25]. The anterior, middle, and posterior ITs converge near the AVN, dividing into two parts: a small portion of the AIT and MIT fibers, along with a large portion of the PIT fibers, enter the lower part of the AVN; the majority of AIT and MIT fibers and a small portion of the PIT fibers terminate at the posterior upper edge of the AVN [26]. The ITs consists of myocardial cells, Purkinje fibers, and a few transitional cells characterized by their compact structure, extending from the oval fossa to the AVN through Todaro’s tendon. This cell population is confined to the left atrial septum [27].

## 4. Manifestations of Dysfunction in Internodal Tracts

**Fig. 3.**
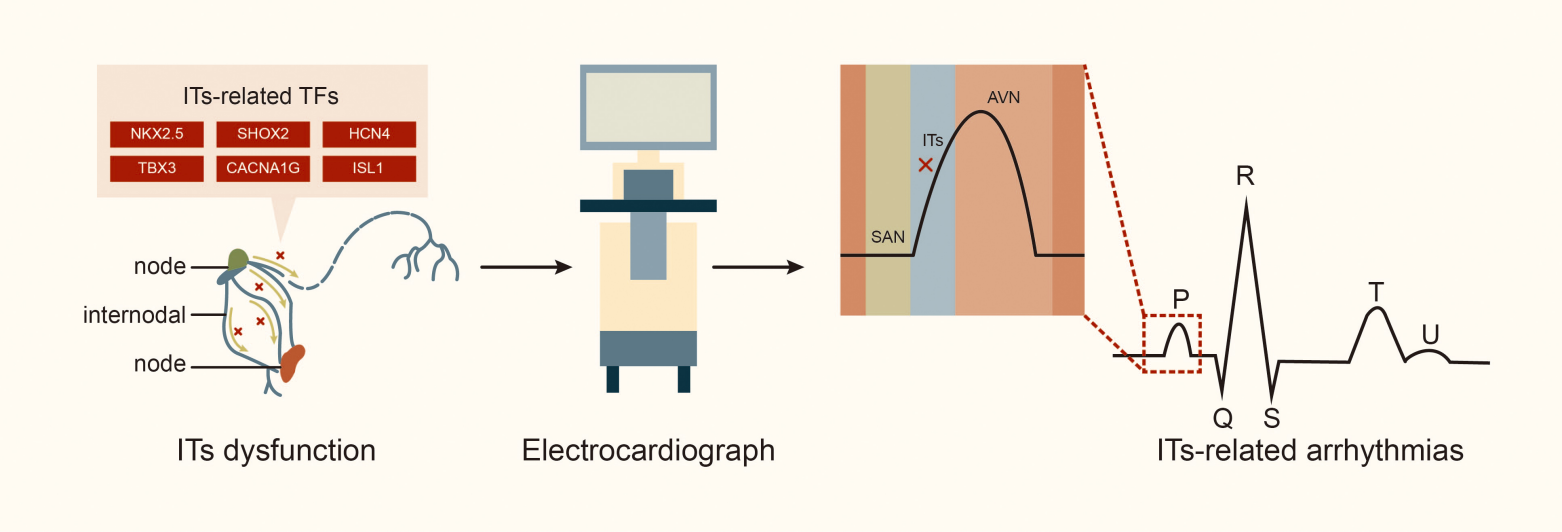
**Internodal tracts dysfunction and related transcription factors**. Abbreviations: TFs, transcription factors; STBX3, T-box transcription factor 3; HCN4, hyperpolarization activated cyclic nucleotide gated potassium channel 4; SHOX2, SHOX homeobox 2; NKX2.5, NK2 homeobox 5; lSL1, ISL LIM homeobox 1; CACNA1G, calcium voltage-gated channel subunit alpha1 G.

The ITs play a crucial role in the CCS by rapidly transmitting electrical signals from SAN to left atrium and AVN [4]. Generally, electrical impulse generated by the SAN is transmitted through the AIT, MIT and PIT (including the BB) to the AVN and the left atrium. Thus, blockage or damage to the ITs can result in internodal or interatrial conduction block, which is associated with electromechanical dysfunction of the left atrium and initiation of various atrial arrhythmias [28, 29, 30]. In an experiment with dogs, Bachmann [31] first described damage to the apical muscle bundle in the atrial septum connecting the left and right atrial appendages, resulting in interatrial conduction block. Waldo *et al*. [32] produced surgical lesions in the left and right atria portions of the BB in the canine heart, and subsequently, delayed conduction of the BB led to partial interatrial block (IAB) according to electrocardiogram observations, while a complete block of the BB led to advanced IAB. Studies indicate that anatomic sites associated with the occurrence of atrial arrhythmia include the atrial septum (where the MIT is located) and crista terminalis (CT, where the PIT runs through) in adult hearts. These areas are critical for transmitting electrical signals from SAN to AIT in the left atrium [6, 14]. A study of the Finnish national population, including 8028 individuals aged 30 and older (of whom 6354 underwent health examinations, including electrocardiograms, and were followed for 15 years) [33], found that approximately 10.7% of patients had symptoms of IAB; the high incidence of IAB in the population suggests that the number of people with ITs conduction disorder may be underestimated. Furthermore, interatrial conduction block is a risk factor for conditions such as supraventricular arrhythmia (including AF), stroke, thromboembolism, sinus node dysfunction, and transient ischemic attacks [33, 34]. Additionally, about half of patients have a history of recurrent paroxysmal AF or atrial flutter, and 40% may experience atrial premature beats and atrial tachycardia [27, 35, 36]. The Wenckebach bundle is associated with the induction of AF, while the unique electrophysiological characteristics of the BB have been observed in patients with AF [14]. In patients with atrial septal defect, the maze procedure can lead to atrial conduction block and AF due to blockage of the AIT and MIT by the defect, while the right lower atrial incision and intermediate septal incision block the PIT [37]. In a study exploring the relationship between damage to the internodal conduction pathway and supraventricular arrhythmia in dogs, conduction time and synchronous activation sequences from the SAN to the vicinity of the AVN were measured using endocardial mapping. The study found that injury to the AIT during cardiopulmonary bypass surgery, regardless of location or extent, significantly prolonged the conduction time from the SAN to the vicinity of the AVN by approximately 15 milliseconds. Additionally, separation of the AIT and PIT, or all the three tracts, can lead to sinus arrhythmias. About 50% of dogs also experience bradycardia, including atrioventricular nodal arrhythmias [38], highlighting the crucial role of internodal tracts in maintaining normal cardiac conduction function (Fig. 3).

Studies have also found that atrial muscle contraction ceases in a high potassium ion environment, and the P wave on the electrocardiogram disappears; however, the ITs continue to transmit impulses from the SAN to the AVN and maintain contractions [25]. Except for the ITs, electrical signal transmission in other atrial cells is completely lost [39], indicating that ITs have greater resistance to high potassium ion environments than general atrial cardiomyocytes.

## 5. Transcription Factors and Internodal Tracts

Research has identified that TFs such as NKX2.5 [17], SHOX2 [40, 41], HCN4 [42], and TBX3 [16] are expressed in the ITs. Additionally, calcium voltage-gated channel subunit alpha1 G (CACNA1G) and ISL1 are crucial in the development of CCS (Table 1, Ref. [17, 41, 42, 43, 44, 45, 46]).

**Table 1.** **The discovery of transcription factors possibly associated with ITs and their regulatory function**.

Author	Year	Transcription factors of ITs	Coding gene	Function of transcription factors
Nakashima *et al*. [17]	2014	NKX2.5	*Nkx2–5*	Inhibits ITs morphogenesis and conductivity
Sun *et al*. [41]	2013	SHOX2	*Shox2*	Regulates the development and maintenance of ITs
Vicente-Steijn *et al*. [42]	2011	HCN4	*Hcn4*	Maintains normal rhythm and regulates ITs development
Soufan *et al*. [43]	2004	TBX3	*Tbx3*	Prevents ITs from developing into ventricular and atrial myocardium
Baudot *et al*. [44]	2020	CACNA1G	*Cacna1g*	Increases the speed of conduction from SAN to ITs
Cai *et al*. [45]	2003	ISL1	*Isl1*	Regulates the ITs development and function
Ye *et al*. [46]	2015	NKX2.5 and SHOX2	*Nkx2–5* and *Shox2*	Co-regulates the cell fate of SAN and ITs

### 5.1 NKX2-5 and Internodal Tracts

*NKX2-5* is a member of the NK2 family of homeobox genes in humans and is homologous to the* tinman* gene found in Drosophila melanogaster. Furthermore, NKX2-5 plays a critical role in cardiac morphogenesis by regulating transcription of various genes involved in this process [47]. Clinically, *NKX2-5* haploinsufficiency is associated with progressive heart defects, such as atrioventricular block, atrial septal defect, and ventricular septal defect [48]. Studies knocking out the *Nkx2-5* gene in mice have shown that the loss of *Nkx2-5* leads to severe cardiac developmental abnormalities, resulting in death around embryonic day 10.5 (E10.5). This underscores the essential role *NKX2-5* has in cardiac structure development. However, the precise role of *Nkx2-5* in the CCS remains poorly understood, partly due to the premature death of *Nkx2-5* knockout mice, which hinders the analysis of tissue development in the SHF [49]. Research on atrial *Nkx2-5* knockout mice [17] revealed three *Hcn4*-positive ITs between the SAN and the AVN. Additionally, *Nkx2-5* knockout led to a significant increase of the *Hcn4*-positive area and thickening of these tracts, whereas the *Hcn4*-positive ITs in the control group were very thin. In the *Nkx2-5* conditional knockout and control groups, Connexin 40 (*Cx40*) in the ITs was negative, indicating that these tracts only maintained slow conductivity. Mice with atrial-specific deficiency of *Nkx2-5* frequently exhibit major atrial abnormalities, such as atrial septal defects (ASDs), supraventricular bradycardia, and atrial conduction block. These abnormalities are attributed to the proliferation of myocardial working cells and conduction cells. Genetic analysis indicated that Notch signaling promotes atrial myocardium proliferation in mouse embryos, while transcriptome analysis showed that *Nkx2-5* inhibits Notch activity in the atrium [17]. These findings suggest that *Nkx2-5* and Notch signaling work together to regulate the proliferation of atrial working and conduction cardiomyocytes. Thus, transcription factor NKX2.5 plays an inhibitory role in the morphogenesis and conductivity of ITs, and they help to prevent the development of these tracts into atrial myocardial tissue by inhibiting Notch signaling. Therefore, NKX2.5 is crucial for both cardiac structure formation and normal function of ITs.

### 5.2 SHOX2 and Internodal Tracts

SHOX2 is a homologous domain transcription factor that promotes the activation of *short stature homeobox 2* (*Shox2*) gene, a member of the homeobox gene family known as *SHOX2* in humans. Mouse *Shox2* and human *SHOX2* share up to 99% homology in amino acid sequences [50]. SHOX2 is a key regulatory factor in the development of CCS, participating in heart rate regulation, and is genetically related to atrial arrhythmias. Its deficiency can lead to bradycardia [51]. Researchers also confirmed that the *SHOX2* gene in humans is a susceptibility gene for AF [52, 53]. In humans, *SHOX2* is located on chromosome 3q25.32 and encodes three protein isoforms: isoform a (331 amino acids), isoform b (355 amino acids), and isoform c (319 amino acids). Previous research has established that SHOX2 regulates the expression of many cardiac genes through transcription, including *Hcn4*, *Nkx2-5*, *Cx40*, Connexin 43 (*Cx43*), natriuretic precursor peptide A (*Nppa*), *Isl1*, and bone morphogenetic protein 4 (*Bmp4*) [54, 55]. Meanwhile, findings by Wang *et al*. [56] show that paired-like homeodomain transcription factor 2 (*Pitx2*) upregulates the *miR-17-92/106b-25* cluster, which inhibits *Shox2*, leading to restricted sinus node development. The absence of this miRNA cluster in mice results in sinus node dysfunction, prolonged PR interval, and increased susceptibility to AF. Studies have also found that AF patients carrying the *SHOX2* 3^′^-UTR c.*28T>C variant (where thymine at the 28th base in the 3^′^ non-coding region of the *SHOX2* gene is replaced by cytosine) have significantly longer PR intervals compared to patients with wild-type alleles [52]. Additionally, patients with the c.580C>T variant (where thymine replaces cytosine at the 580th base) may produce a p.R194X truncated protein (with only 193 amino acids), leading to sinus bradycardia, as well as a significantly prolonged PR interval, RR interval, and QRS wave [53]. These studies indicate that missense mutations in both coding and non-coding regions of the *SHOX2* gene can significantly reduce or even eliminate *SHOX2* expression, and the resulting arrhythmia symptoms can be stably inherited. *Isl1*, a specific marker for the second cardiac region, is activated by wild-type *Shox2* during transcription. In addition, the *Shox2* R194X mutant mice fail to activate* Isl1*, demonstrating a direct transcriptional connection between *Shox2* and the ITs [53, 57]. In mouse models, homozygous *Shox2* deletion leads to increased mortality between E11.5 and E13.5 due to severe cardiovascular defects, including bradycardia, underdeveloped sinus node and valve, and abnormal expressions of *Cx40*, *Cx43*, and *Nkx2-5*. The mortality rate can be mitigated with closely related hypomorphic *Shox* allele replacing *Shox2*, however the arrhythmias persist [19, 58]. Early in embryonic cardiac development, *Shox2* is expressed in the SAN, right venous valve, and dorsal mesenchymal protrusion of the heart [19, 40]. Sun *et al*. [40, 41] have also observed *Shox2* expression in the ITs, which share an origin with the right venous valve. Conditional knockout of *Shox2* in mice results in incomplete development of the SAN junction and the MIT and PIT, causing symptoms such as atrial block and bradycardia in adult mice. Further, *Hcn4* expression in the AIT disappears while the AIT morphology remains unaffected, suggesting potential alterations in ITs’ conductivity. These findings indicate that the deletion of the *SHOX2* gene or a mutation may lead to defects in the morphology, development and function of the SAN or ITs. SHOX2 is thus a critical factor in regulating the development and maintenance of the entire CCS, especially the SAN and the ITs.

### 5.3 HCN4 and Internodal Tracts

HCN4 is a subtype of hyperpolarization-activated cyclic nucleotide-gated (HCN) channels, acting as a pacing channel that is highly expressed during sinus node development. Studies have shown that *Hcn4* is a marker for the first cardiac region and cardiac pacing cells. During different stages of cardiac development, *Hcn4* is dynamically expressed in various differentiated cardiomyocyte precursors, including those in the CCS that produce different components essential for cardiac function [10]. In studies on chicken embryonic CCS development, *HCN4* expression was found to be restricted to the SAN, ITs, AVN, bundle branch, and atrioventricular annulus myocardium, making it as a CCS development marker in chickens [42]. Meanwhile, changes in HCN4 channel function are associated with sinus node dysfunction and arrhythmias such as AF, ventricular tachycardia, and atrioventricular block [59]. Complete or heart-specific ablation of *Hcn4* in mice impairs embryonic development, resulting in death between E9.5 and E11.5 [60]. Cardiac specific *Hcn4 *knockout mice exhibit severe bradycardia and atrioventricular block, leading to cardiac arrest and death [61], highlighting the critical role of *Hcn4* in maintaining normal rhythm. Thus, the expression pattern of *HCN4* during development may indicate potential arrhythmia targets in hearts and has connections with the development and function of the ITs.

### 5.4 TBX3 and Internodal Tracts

The T-box factor is part of an ancient protein family consisting of evolutionarily conserved transcription factors crucial for embryonic development. T-box 3 (*Tbx3*), a member of this family, is expressed in various tissues and plays a key regulatory role in organs such as the heart, breast, and limbs [62]. Soufan *et al*. [43] have identified TBX3 as a transcription inhibitor in the internodal region. *Tbx3* is continuously expressed throughout cardiac development from the SAN to the atrioventricular region, starting from E8.5. The expression regions coincide with regions showing higher levels of *Nppa* and *Cx40*, which are markers of faster conduction velocity [63, 64, 65]. The peripheral ventricular conduction network, marked by rapid conduction and expression of ventricular-specific myocardial markers, contrasts with the CCS, which features slower conduction and lower expression of these markers [20, 65]. *Tbx3* and *Tbx2* share structural and functional similarities, including inhibitory characteristics and DNA recognition elements [66]. From early embryonic stages, *Tbx2* expression decreases while *Tbx3* expression increases in the heart. Development of the CCS occurs within the *Tbx2* expression regions, where TBX2 inhibits ventricular formation by suppressing ventricular type-specific myocardial markers *Nppa* and *Cx40* [67, 68]. This indicates that the CCS develops in regions where ventricular formation is inhibited. In another three-dimensional reconstruction study on mouse heart development, *Tbx3* was selectively expressed throughout the CCS, including in the ITs [43]. TBX3 inhibits the promoter activity of *Nppa* and *Cx40* and blocks the synergistic activation of the *Nppa* promoter by TBX5 and NKX2.5, thus contributing to the sustained inhibition of ventricular phenotype development in the CCS-derived myocardium [16]. Additionally, TBX3 has been shown to bind and inhibit atrial genes, such as the *Cx43* gene, thereby suppressing the working atrial myocardial phenotype [69]. These findings suggest that the TBX3 transcription factor plays a crucial role in the development of ITs and may prevent their differentiation into the ventricular or atrial myocardium.

### 5.5 CACNA1G and Internodal Tracts

Recent studies have confirmed the presence of the CACNA1G transcription factor in the CCS. *CACNA1G* encodes the pore-forming subunit of the low-voltage activated T-type calcium channel (Ca_v_3.1) channel, which is expressed in various regions of the central nervous system, particularly in Purkinje neurons and deep cerebellar nuclear. The Ca_v_3.1 channel is characterized by low activation voltage, relatively small single-channel conductivity, and rapid deactivation [70]. Further, the Ca_v_3.1 channel is believed to be associated with pacemaker activity, low-threshold calcium spikes, neuronal oscillation, resonance, and rebound pulse discharge. Although Ca_v_3.1 is expressed in the embryonic hearts of various mammals, its expression decreases with the progress of development [71]. The functional role of Ca_v_3.1 has been confirmed in rodents; mice lacking Ca_v_3.1 channels exhibited reduced pacemaker activity and worsened atrioventricular conduction compared to wild-type mice [72]. Ca_v_3.1 mRNA and protein have also been detected in the CCS of humans [73]. Baudot *et al*. [44] demonstrated that *Cacna1g* expression is linked to the autonomy of pacemaker cells, whereby increased *Cacna1g* expression correlated with increased conduction velocity from the SAN to the ITs while pacing conduction velocity decreased upon reaching the AVN. This suggests that the ITs are the dominant conduction pathway from the SAN to the AVN and may be associated with atrioventricular delay.

### 5.6 ISL1 and Internodal Tracts

The ISL1 transcription factor is crucial for the development of the CCS. The *Isl1* gene encodes a transcription factor with a LIM homeodomain. *Isl1* is expressed in adult mammalian cardiac stem cells and progenitor cells, primarily in the outflow tract, right ventricle, and parts of the atrium. This area, known as the second cardiac region, is considered a convergence place for cardiac progenitor cells; therefore, deletion of this gene results in abnormal atrioventricular formation [45]. Homozygous *Isl1* mutants exhibit growth retardation around E9.5–10 and death around E10.5–11. Histological analysis showed severe cardiac developmental defects in these mutants, including the absence of the right ventricle and outflow tract and a common atrium [45]. Additionally, *Isl1* is a direct transcriptional target of *Shox2*, which can rescue *Shox2*-mediated bradycardia [57]. During SAN development, *Shox2* regulates *Isl1*expression, suggesting that *Isl1* is crucial for SAN development and may influence ITs’ development and function, potentially impacting atrioventricular delay.

### 5.7 Potential Co-regulation of Internodal Tracts by SHOX2 and NKX2.5

Research by Ye *et al*. [46] on the SAN revealed that it can be divided into two distinct regions: the *Shox2^+^/Hcn4^+^* SAN head and the *Shox2^+^/Nkx2-5^+^/Hcn4^+^* SAN junction. The specific knockout of *Shox2* at the SAN junction results in an underdeveloped SAN junction and ectopic expression of the working myocardial marker *Cx40* in cells at this junction. This indicates that *Shox2 *knockout can cause a shift of cell fate from SAN cells to working myocardial cells. Additionally, an intriguing antagonistic interaction between *Shox2 *and *Nkx2-5* was observed. Conditional knockout of the *Shox2* gene at *Nkx2-5* expression sites and subsequent reduction of *Nkx2-5* expression partially reversed the SAN junction defects caused by *Shox2* knockout, allowing for the re-expression of *Hcn4*, *Tbx3*, and *Isl1* at the SAN junction. This suggests a reversion of cell fate from working myocardial cells to *Shox2^+^/Nkx2-5^+^* pacemaker cells. These findings highlight how the *Shox2–Nkx2-5* antagonistic mechanism can alter cell fate in the SAN and regulate its morphology. Given that the morphogenesis of both the ITs and the SAN junction starts simultaneously from the second cardiac region [10, 11, 74], it is reasonable to hypothesize that the ITs are also co-regulated by *Shox2* and *Nkx2-5*.

Furthermore, mutations in the human Notch and Ras-MAPK (mitogen-activated protein kinase) signal pathways are crucial for myocardial cell proliferation and can lead to atrial block and atrial septal defect (ASD) [75]. Indeed, it was confirmed that *Shox2* is a target gene of the pituitary homeobox gene (*Pitx2*) [56]. Meanwhile, one *Pitx2* subtype, *Pitx2c*, directly binds to and inhibits *Shox2* expression. Haploid dysfunction of *Pitx2c* predisposes mice to atrial arrhythmias [69], suggesting that Notch, Ras-MAPK signal pathways and *Pitx2* may play roles in the developmental regulation of internodal tracts.

## 6. Expectations

As a part of the CCS, the ITs are extremely important for studying cardiovascular diseases, especially in exploring the pathogenic mechanisms of congenital or acquired heart conduction system-related diseases. However, there are still limitations in current understanding, with limited reports on the existence, morphogenesis, developmental process, and function of ITs. Firstly, Ye *et al*. [46] found a mutually antagonistic molecular mechanism between *Nkx2-5* and *Shox2*, which has potential regulatory effects on the morphogenesis of SAN junction and the fates of working myocardial cells and pacemaker cells. As the morphogenesis process of the SAN junction and ITs begin simultaneously and both originate from the second cardiac region, it is reasonable to speculate that *Nkx2-5* and *Shox2* may jointly regulate ITs. However, more experiments are still needed for proof. Secondly, the regulatory mechanisms of transcription factors such as HCN4, TBX3, and PITX2 on the morphogenesis and function of ITs have not been fully elucidated, and the relationship between *Hcn4*, *Tbx3*, *Nkx2-5*, *Shox2* and the regulation of ITs still needs further exploration. Thirdly, transcription factors such as CACNA1G and ISL1 are closely related to the development of CCS and may also be involved in the regulation of ITs’ development and function. Lastly, other complex molecular mechanisms may exist in the regulation of ITs, which require further exploration.

## 7. Conclusions

Although significant progress has been made in the study of ITs, the developmental mechanisms, formation processes, and specific functions of these tracts still need to be better understood, particularly the regulatory mechanisms, where various research views exist. Research on ITs has reshaped our understanding of CCS and offers a theoretical foundation for the onset of certain arrhythmias. However, the exact role of ITs in CCS and their contributions to the pathogenesis of cardiovascular diseases, especially arrhythmias, remains to be fully elucidated. In conclusion, ITs warrant increased attention, and further research investment is essential to advance our understanding of this crucial apparatus, ultimately driving progress in cardiology and clinical medicine.

## Abbreviations

AIT, anterior internodal tract; CCS, cardiac conduction system; ITs, internodal tracts; MIT, middle internodal tract; PIT, posterior internodal tract; SAN, sinoatrial node.
